# Etiology Distribution, Clinical Characteristics, and Suboptimal Pacing Outcome of Atrioventricular Block in Young Patients

**DOI:** 10.31083/j.rcm2409250

**Published:** 2023-09-06

**Authors:** Zhongli Chen, Yuanhao Jin, Nan Xu, Yuan Gao, Sijin Wu, Yan Dai, Keping Chen

**Affiliations:** ^1^State Key Laboratory of Cardiovascular Disease, Cardiac Arrhythmia Center, Fuwai Hospital, National Center for Cardiovascular Disease, Chinese Academy of Medical Sciences and Peking Union Medical College, 100037 Beijing, China; ^2^Department of Echocardiography, Fuwai Hospital, National Center for Cardiovascular Diseases, Chinese Academy of Medical Sciences and Peking Union Medical College, 100037 Beijing, China

**Keywords:** atrioventricular block, etiology, young, pacing

## Abstract

**Background:**

The causes of atrioventricular block (AVB) are different and diverse young patients, as compared to the old. However, little is known about the etiology distribution and clinical characteristics of AVB in the young group.

**Methods:**

We retrospectively analyzed clinical information for AVB patients under 50 years of age. We summarized clinical phenotypes for patients with undetermined AVB etiology, according to AVB type and cardiac-structural change, whereas those who received pacing therapy were followed up for suspected heart failure events (HFEs).

**Results:**

AVB etiology was identified in only 289 (61.4%) patients, while 38.6% still have undertermined etiology for AVB. Non-ischemic cardiomyopathy (16.6%) and complication of cardiac surgery (13.4%) were the top two etiologies. In addition, four distinct phenotypes were identified in AVB patients with undetermined etiology, of which the severe phenotype (both borderline/elevated left ventricular diameter or abnormal left ventricular ejection fraction and advanced AVB) accounted for 17%. Notably, 80.7% of patients with severe phenotype received pacing therapy. Based on a median follow-up time of 17.5 months, we found the occurrence of 16 suspected HFEs in 110 pacemaker receivers (12 were lost to follow up). Notably, the severe phenotype was associated with a higher risk of heart failure (HF) symptoms.

**Conclusions:**

AVB etiology in young patients under 50 years of age is complex and underdiagnosed. In patients with undetermined etiology, severe phenotype featuring advanced AVB and abnormal Left ventricle (LV) structure/function is associated with a higher rate of HF symptoms even after pacing therapy.

## 1. Introduction 

Atrioventricular block (AVB) is a common and significant cardiac conduction disorder, with a high rate of pacemaker implantation [1, 2]. Previous studies have reported higher morbidity and mortality rates in elderly AVB patients, mainly due to the occurrence of idiopathic aging-related fibrosis of the cardiac conduction system [3, 4]. For decades, pacing has been considered the “one size fits all” approach for alleviating AVB symptoms [5]. Early AVB onset cannot be ignored in the young population, although its occurrence is rare; a previous study reported prevalence rates of 2.67‰ and 6.23‰ in Chinese patients aged 18–39 and 40–59 years, respectively [6]. Evidence from western countries has demonstrated that the etiologic spectrum of AVB is more diverse in the young population, and may range from congenital heart disease (CHD) to rare cardiomyopathy [7, 8, 9]. Premature AVB in young patients can affect their productive years due to the probable longer disease courses, as well as the economic burden associated with medical treatment. Nevertheless, pacing does not achieve as excellent a prognosis in the young population as it does in older patients. Findings from a large-scale cohort study revealed that younger patients had a higher relative risk of heart failure (HF) hospitalization than did their older counterparts, which might be attributed to a synergistic effect of the progression of underlying etiologies and long-term high right ventricular pacing (RVP) rate [10, 11]. However, the clinical decision for AVB treatment is mainly made based on the AVB blocking site, with little attention paid to the underlying causes of the condition [1].

Determination of presumed AVB etiology in young patients is also necessary for a deep understanding of the underlying disease mechanism, timely assessment of an individual’s prognosis, and appropriate application of treatment therapies [12]. To date, however, early-onset AVB remains underappreciated with no integrated descriptions of etiology and clinical characteristics, especially in the Chinese population. In the present study, we report etiologic distribution and clinical characteristics of AVB patients younger than 50 years, and evaluate the relationship between etiology, blocking type, and cardiac structural/functional change to provide new insights about AVB in the young group.

## 2. Materials and Methods

### 2.1 Study Sample and Selection Criteria

This single-center retrospective study included AVB patients aged ≤50 years, who were admitted to the National Center for Cardiovascular Disease at the Fuwai Hospital in China between September 2019 and March 2022. Patients were included in the study if they were diagnosed at our hospital with AVB as confirmed on the 12-lead surface and/or telemetry/Holter monitoring, and were ≤50 years old. To avoid duplication, we included the first hospitalization episode of AVB diagnosis. We also collected each patient’s demographics, onset symptoms, comorbidities, relevant investigations (e.g., 12-lead electrocardiogram [ECG], laboratory biomarkers, echocardiography findings), and any treatment with a cardiac implantable electronic device (CIED). Patients were excluded if they lacked medical records, or had missing data on important parameters such as medical history, ECG, laboratory test, or echocardiography information. The study was approved by the Ethics Committee of Fuwai Hospital (IRB Approval NO. 2022-1788), and informed consent was obtained from all participants.

### 2.2 Review Process

Two trained physicians independently performed in-depth reviews of the medical records, based on a pre-defined etiologic classification. Any disagreement between them, with regard to etiologic determination, was adjudicated by a senior consultant. Congenital AVB (CAVB) was considered etiology if AVB was diagnosed in utero, at birth, or within the first month of life, in addition to any evidence of abnormal maternal anti-SSA/Ro-SSB/La antibodies [13]. CHD was considered etiology if AVB occurred in the setting of significant cardiac defects, including AV septal defects, secundum atrial septal defects, ventricle defects, Steno-Fallot tetralogy, transposition of the great arteries, or univentricular heart anatomy [14, 15]. On the other hand, etiology was registered as hereditary if both the familial trend and a proven pathogenic gene mutation were identified. AVB was considered a complication of cardiac surgery (including transcatheter aortic valve replacement) or intracardiac ablation, if there was no evidence of AVB prior to the operation but there was within 30 days after operation [16, 17]. AVB cases with a confirmed diagnosis of non-ischemic cardiomyopathy (NICM), infiltrative cardiomyopathy, neuromuscular disease, a confirmed history of myocarditis, Long QT (LQT), Brugada syndrome (BrS), or immune disease (systemic sclerosis, Sjogren’s syndrome, systemic lupus erythematosus, reactive arthritis, or other auto-immune diseases) were all classified as etiology [3, 18, 19, 20, 21]. Endocrine-related AVB was registered if a patient presented with thyroid dysfunction that was resolved after corrected thyroid function [22]. Ischemic heart disease (IHD) was considered etiology if patients developed AVB due to myocardial infarction or were found to have AVB during angina. Drug-related AVB was considered etiology if AVB occurred during therapy with a calcium channel blocker, digoxin, beta-blockers, or antiarrhythmic drugs, but was resolved and did not recur after discontinuation of the drug [23]. Vagal mediation was diagnosed if AVB was associated with high vagal tone, such as during sleep, accompanied by slowing of sinus rhythm documented during a tilt test [24].

### 2.3 Measurements and Definitions

Baseline echocardiography was performed in the same laboratory and reviewed by an experienced sonographer. The left atrium dimension was measured in the anterior-posterior plane, at the level of aortic sinuses, whereas the left ventricular ejection fraction (LVEF) was measured via unenhanced 2D echocardiography using the modified Simpson biplane method. The diameter of right ventricle (RV) was measured at the mid-level of the RV. Blood samples were collected from each patient 1–3 days after admission, and creatinine kinase and N-terminal B-type natriuretic peptide (NT-proBNP) were directly measured using a commercial chemiluminescence assay (Roche Diagnostics GmbH, Mannheim, Germany), according to the manufacturer’s instructions. Borderline or elevated left ventricle diastolic diameter (LVEDD) was defined if the value exceeded 55 mm for an adult male patient, or 50 mm for women and pediatric patients [25]. Abnormal LVEF was defined as a LVEF value of ˂50% for adults or ˂60% for pediatrics. HF with reduced ejection fraction (HFrEF, LVEF ≤40%) and HF with mildly reduced ejection fraction (HFmrEF, LVEF = 41–49%) were defined according to the 2021 European Society of Cardiology guidelines for the diagnosis and treatment of acute and chronic HF [26]. Impaired RV systolic function was described by an abnormally low tricuspid annular plane systolic exclusion (TAPSE), which was defined as TAPSE <17 mm [27]. Advanced AVB was defined as Mobitz Type II AVB, high-degree AVB, or third-degree AVB, which was associated with intra- or infra-Hisian block and pacemaker implantation [1]. Left ventricle (LV) structural/functional change is defined as LVEF <50% (abnormal LVEF) or borderline or elevated LVEDD. The severe phenotype was defined as advanced AVB concomitant with abnormal LVEF/borderline/elevated LVEDD.

### 2.4 Follow-Up and Clinical Outcomes

Patients with undetermined AVB causes and receiving pacing therapy were followed up via outpatient review or telephone by physicians, for suspected HF events (HFEs) defined as new onset of HF-related symptoms and signs, unplanned clinic visit, or hospitalization due to the symptoms. Specifically, we measured the duration from pacemaker implantation to the latest follow-up time, as well as the time to event or loss to follow-up, for each patient. The target HF-related signs and symptoms included breathlessness, fatigue, depression, and decreased exercise tolerance, as well as symptoms of volume overload, such as swelling of the legs or increase in abdominal distension, as previously described [28, 29]. The echocardiographic results during follow-up at either our outpatient clinic or the local hospital were also recorded if avialiable.

### 2.5 Statistical Analysis

Conservatively calculating, a sample size of 503 produces a two-tailed 95% confidence interval with a width equal to 0.100 when considering an etiology proportion of 50% and 20% of records with missing data. Continuous variables were expressed as mean (standard deviation) if they were normally distributed, otherwise, they were presented as median (interquartile range). Categorical variables were presented as numbers and proportions. Proportions were compared using the Chi-Square or Fisher exact probability test, as appropriate. Comparisons between two groups of continuous variables were performed using a Students’ *t*-test, analysis of variance (ANOVA), Mann–Whitney U test, or Kruskal-Wallis test, as appropriate. We also generated Kaplan-Meier survival curves with a log-rank test to compare event-free survival rates between patients with severe phenotype and non-severe phenotype in an unknown etiology group with pacing therapy. Univariate and multivariate analyses were conducted using Cox proportional hazard model and binary logistic regression model. Statistical tests were two-tailed, with *p* < 0.05 considered statistically significant. All analyses were performed using IBM SPSS Statistics 22 (IBM Corp., Armonk, NY, USA) and packages contained in R version 4.1.2 (R Foundation for Statistical Computing, Vienna, Austria).

## 3. Results

**Fig. 1.**
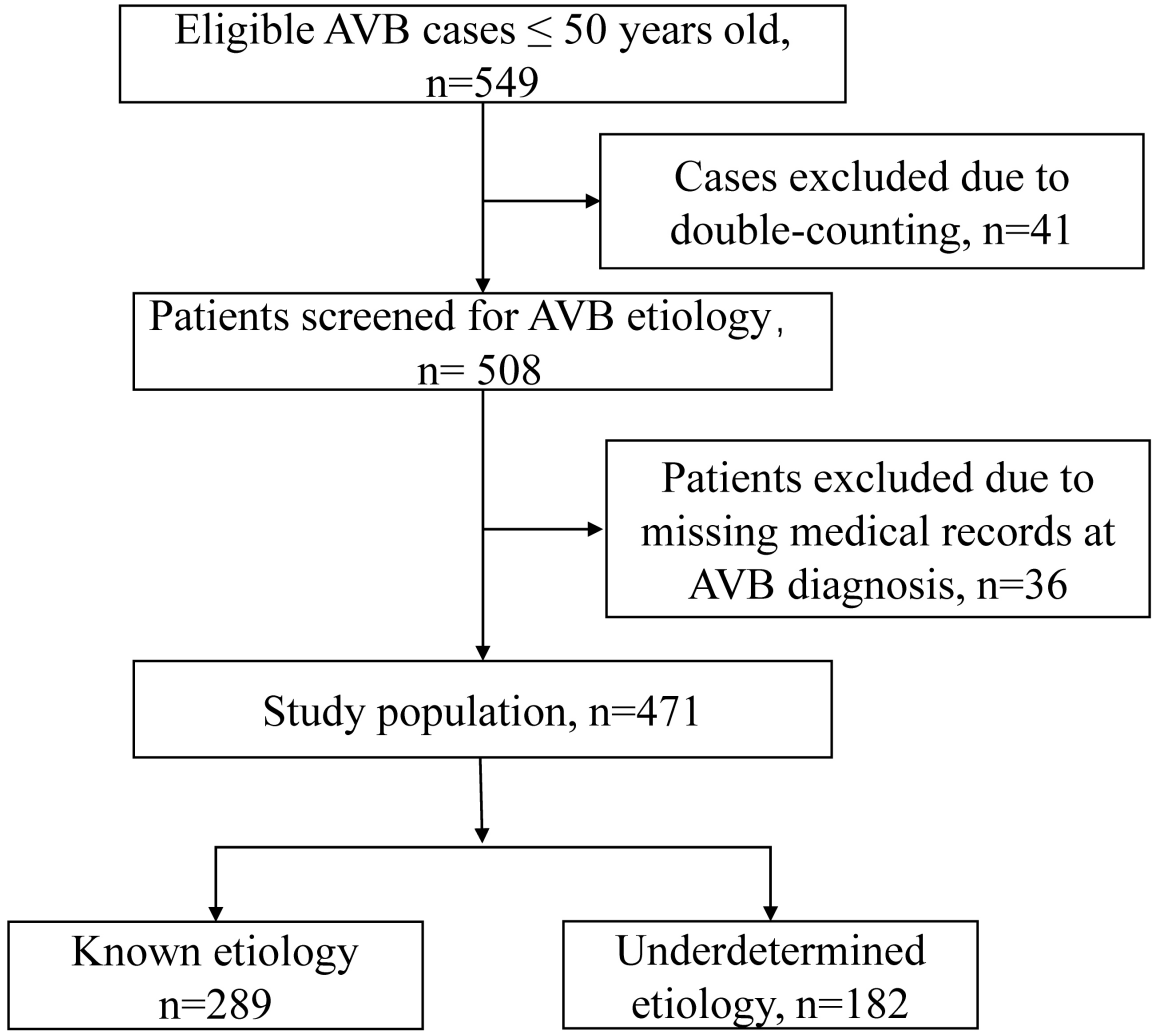
**Flowchart for inclusion of inpatients with the atrioventricular block below 50 years old**. AVB, atrioventricular block.

Among the initial 549 eligible AVB cases aged ≤50 years, 41 were excluded due to duplication brought about by readmission during enrollment. Another 36 were excluded due to missing medical records, ECG, echocardiography, or negative results for cardiac biomarkers at AVB diagnosis. A total of 471 patients were eligible for final analysis, of whom the mean age was 34.1 years; 291 (61.8%) were male (Fig. 1). Advanced AVB cases comprised 67.7% of the study sample, in which Mobitz II, high-degree AVB, and third-degree AVB accounted for 14.7%, 16.6%, and 68.7%, respectively. The most commonly observed symptoms included chest tightness (29.3%), palpitation (28.7%), and dizziness (20.4%). The most prevalent comorbidities in this younger population were valve disease (26.5%), systemic arterial hypertension (17.2%), and pulmonary artery hypertension (13.8%). Moreover, 15.9% of the sample exhibited atrial fibrillation or flutter, whereas 15.5% also had right-bundle-branch block. A CIED was implanted in 258 (54.8%) patients, of whom 11 underwent cardiac resynchronization therapy (CRT) whereas 9 received an implantable cardiac defibrillator. Among the 238 patients who received single- or dual-chamber pacemakers, 30.3% and 69.7% had His-Purkinje system pacing, and traditional RVP, respectively (Table 1). Of note, there are 66 (14%) pediatric patients, including 5 infants, 8 toddlerhooder, 8 in early childhood, 8 in middle childhood and 37 in early adolescence according to National Institute of Child Health and Human Development (NICHD) Pediatric Terminology. Among these pediatric patients, cardiac-surgery related AVB was dominate etiology (37.9%), while 33.3% were underdiagnosed. And 46 patients were in advanced AVB, with 80.4% (n = 37) of them received pacing therapy (**Supplementary Table 1**).

**Table 1.** **Clinical characteristic of young and middle-aged AVB patients**.

			Total (n = 471)	Known reason (n = 289)	Unknow reason (n = 182)	*p*-value
Age, yrs			34.1 ± 12.9	33.5 ± 13.5	35.0 ± 11.8	0.192
Male sex, n %			291 (61.8%)	192 (66.4%)	99 (54.4%)	0.009
BMI, kg/$m^{2}$			23.7 ± 4.9	23.6 ± 5.3	23.7 ± 4.3	0.805
Onset age, yrs			31.0 ± 14.0	30.4 ± 14.6	31.85 ± 13.0	0.273
Family history, n %			33 (7.0%)	21 (7.3%)	12 (6.6%)	0.781
Symptoms						
	Dizziness, n %		96 (20.4%)	45 (15.6%)	51 (28.0%)	0.001
	Chest tightness, n %		138 (29.3%)	90 (31.1%)	48 (26.4%)	0.268
	Palpitation, n %		135 (28.7%)	77 (26.6%)	58 (31.8%)	0.222
	Amaurosis, n %		77 (16.4%)	41 (14.2%)	36 (19.8%)	0.110
	Fatigue, n %		82 (17.4%)	34 (11.7%)	48 (26.4%)	<0.001
	Syncope, n %		88 (18.7%)	48 (16.6%)	40 (22.0%)	<0.001
	Asymptomatic, n %		70 (14.9%)	41 (14.2%)	29 (15.9%)	0.604
Comorbidities						
	Coronary artery disease, n %		63 (13.4%)	53 (18.3%)	10 (5.5%)	<0.001
	Valve disease, n %		125 (26.5%)	106 (36.7%)	19 (10.4%)	<0.001
	Pulmonary artery hypertension, n %		65 (13.8%)	52 (18.0%)	13 (7.1%)	<0.001
	Stroke, n %		15 (3.2%)	11 (3.8%)	4 (2.2%)	0.333
	Vasovagal syncope, n %		12 (2.6%)	9 (3.1%)	3 (1.7%)	0.326
	OSAHS, n %		37 (7.7%)	26 (9.0%)	11 (6.0%)	0.246
	Diabetes, n %		43 (9.1%)	31 (10.7%)	12 (6.6%)	0.129
	Myocarditis, n %		18 (3.8%)	16 (5.5%)	2 (1.1%)	0.014
	Thyroid disease, n %		24 (5.10%)	17 (5.88%)	7 (3.85%)	0.328
	Chronic kidney disease, n %		15 (3.18%)	13 (4.50%)	2 (1.10%)	0.041
	Hypertension, n %		81 (17.2%)	52 (18.0%)	29 (15.9%)	0.564
	NYHA class		1.6 ± 0.9	1.8 ± 1.0	1.2 ± 0.5	<0.001
		I, n %	309 (65.6%)	151 (52.3%)	158 (86.8%)	<0.001
		II, n %	85 (18.0%)	69 (23.9%)	16 (8.8%)	
		III, n %	56 (11.9%)	48 (16.6%)	8 (4.4%)	
		IV, n %	21 (4.5%)	21 (7.2%)	0 (0.0%)	
AVB type						
	Mild AVB, n %		152 (32.3%)	95 (32.9%)	57 (31.3%)	0.726
	Advanced AVB, n %		319 (67.7%)	194 (67.1%)	125 (68.7%)	
		Mobitz type II	47 (14.7%)	26 (13.4%)	21 (16.8%)	
		High degree AVB	53 (16.6%)	28 (14.4%)	25 (20.0%)	
		Third degree AVB	219 (68.7%)	140 (72.2%)	79 (63.2%)	
ECG features						
	Heart rate, bpm		59.0 (43.5–72.5)	62.0 (44.0–74.0)	56.0 (43.0–70.8)	0.042
	Atrial fibrillation/flutter, n %		75 (15.9%)	50 (17.3%)	25 (13.7%)	0.303
	LBBB, n %		37 (7.9%)	25 (8.7%)	12 (6.6%)	0.419
	RBBB, n %		73 (15.5%)	59 (20.4%)	14 (7.7%)	<0.001
	No sustained VT, n %		54 (11.5%)	42 (14.5%)	12 (6.6%)	0.008
Echocardiographic features						
	Left atrial diameter, mm		36.3 ± 8.8	37.2 ± 9.9	34.8 ± 6.6	0.004
	Left ventricular end diastolic diameter, mm		49.6 ± 10.8	49.9 ± 12.7	49.1 ± 6.7	0.446
	LVEF, %		58.3 ± 12.3	55.6 ± 14.3	62.7 ± 6.2	<0.001
	Right ventricular diameter, mm		24.2 ± 6.9	24.8 ± 8.0	23.3 ± 4.3	0.016
	Abnormal TAPSE, n %		46 (9.8%)	42 (14.5%)	4 (2.2%)	<0.001
	Abnormal LVEF, n %		81 (17.2%)	75 (26.0%)	6 (3.3%)	<0.001
	HFrEF, n %		53 (11.3%)	52 (18.0%)	1 (0.6%)	<0.001
	Borderline/elevated Left ventricle, n %		129 (27.4%)	92 (31.8%)	37 (20.3%)	0.006
	NT-proBNP, pg/mL		212.0 (52.8–940.5)	495.0 (103.00–1702.4)	70.2 (26.0–312.6)	<0.001
	CK, IU/L		76.0 (53.0–112.5)	75.0 (50.0–121.0)	79.0 (58.00–105.5)	0.870
CIED implantation			258 (54.8%)	148 (51.2%)	110 (60.4%)	0.050
	CRT (D), n %		11 (2.3%)	9 (3.1%)	2 (1.1%)	0.159
	ICD, n %		9 (1.9%)	8 (2.8%)	1 (0.5%)	0.087
	Single/dual chamber pacemaker, n %		238 (50.5%)	131 (45.3%)	107 (58.8%)	0.004
Pacing site						0.006
	RV pacing, n %		166 (35.2%)	101 (34.9%)	65 (35.7%)	
	His-Pukenje system pacing, n %		72 (15.3%)	30 (22.9%)	42 (39.3%)	

AVB, atrioventricular block; BMI, body mass index; OSAHS, obstructive sleep apnea-hypopnea syndrome; NYHA, New York Heart Association; Mild AVB, 1st-degree or Mobitz type I AVB; Advanced AVB, Mobitz type II or high-degree AVB or third-degree AVB; LBBB, left bundle branch block; ECG, electrocardiogram; RBBB, right bundle branch block; VT, ventricular tachycardia; TAPSE, tricuspid annular plane systolic excursion; LVEF, left ventricular ejection fraction; HFrEF, heart failure with reduced ejection fraction; NT-proBNP, N-terminal B-type natriuretic peptide; CK, creatine kinase; CIED, cardiac implantable electronic device; CRT(D), cardiac resynchronization therapy (defibrillator); ICD, implantable cardiac defibrillator; RV, right ventricle. The continuous parameters were displayed as mean (standard deviation) or median (25%, 75% interquartile).

### 3.1 Etiology Distribution 

**Fig. 2.**
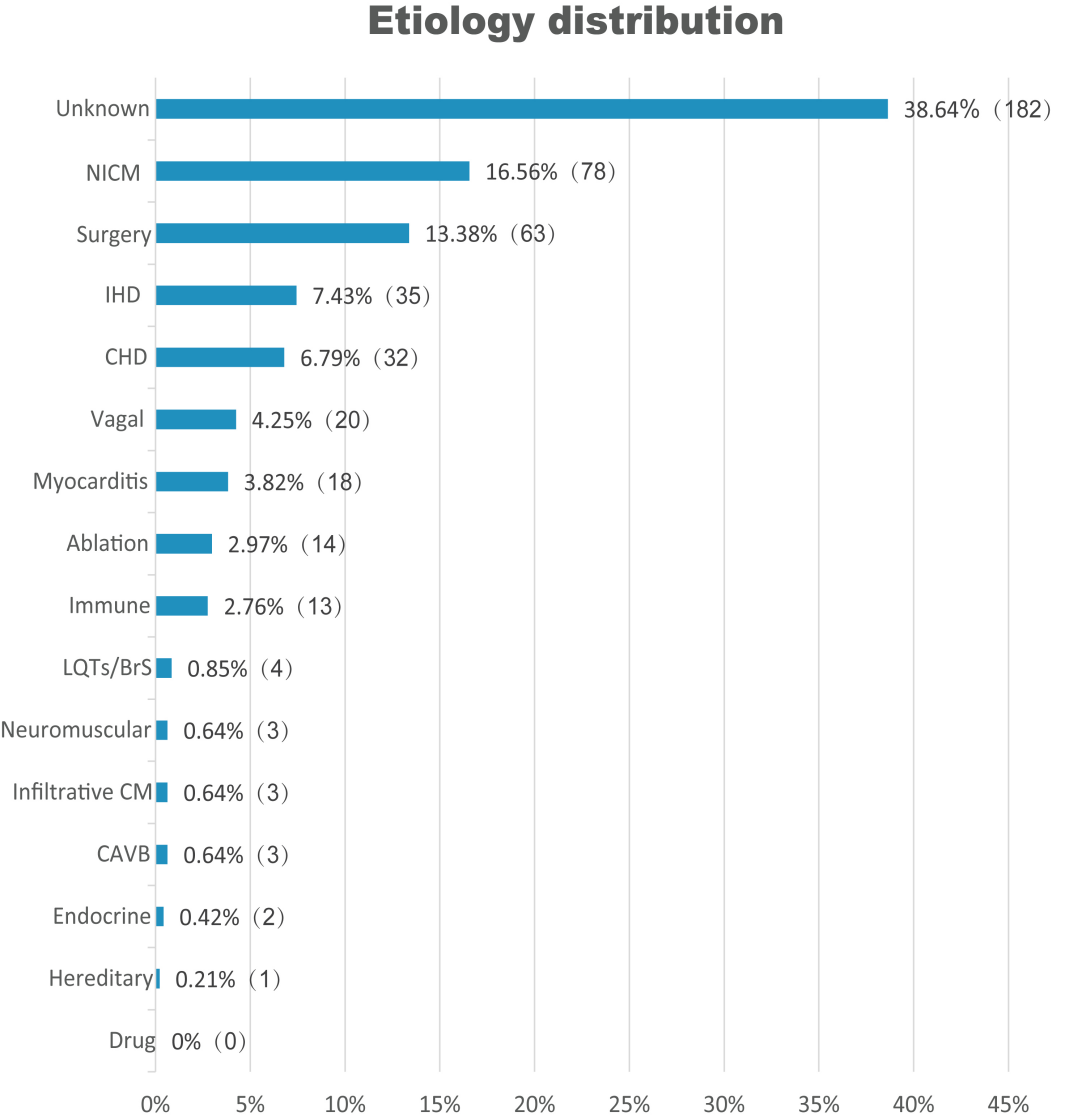
**Etiological distributions of AVB in patients ≤50 years old (n = 471)**. NICM, non-ischemic cardiomyopathy; IHD, ischemic heart disease; CHD, congenital heart disease; LQTs/BrS, long QT syndrome or Brugada syndrome; CM, cardiomyopathy; CAVB, congenital AVB; AVB, atrioventricular block.

As shown in Fig. 2, AVB etiology was identified in only 289 (61.4%) patients. The most commonly known etiology was NICM (n = 78, 16.6%), followed by complications associated with cardiac surgery (n = 63, 13.4%), IHD (n = 35, 7.4%), CHD (n = 32, 6.8%), and vagal-mediated AVB (n = 20, 4.3%). AVB was attributed to myocarditis, complications from ablation, LQTs/BrS, infiltrative cardiomyopathy, and CAVB; endocrine or hereditary causes were in relatively low proportion (overall 12.5%). We found no evidence of any confirmed medication-induced AVB cases in this sample. Notably, no attributable etiology was determined in 38.6% of the patients.

We observed statistically significant differences in the proportions of etiology distribution among different age and sex groups (all *p* < 0.001) (**Supplementary Figs. 1,2**). We stratified the study sample into minors, young adults, and middle-aged adult groups based on cut-offs of 18 and 35 years, then analyzed known etiologies. Although NICM, cardiac surgery, and CHD played an important role in all age-stratified groups, cardiac surgery-related complications were frequently diagnosed in minors, and cardiac surgery was the leading etiology (37.9%). On the other hand, vagal-mediated AVB and ablation-related AVB were more common in young adults than in minors and middle-aged adults. IHD was the most dominant AVB etiology in the middle-age group, but occurred in low proportions in the rest of the groups. Unknown etiology was also a significant component across the three age groups (**Supplementary Fig. 1**). Analysis of sex-specific etiologic patterns revealed that NICM, IHD, neuromuscular disease, and vagal-mediated AVB frequently occurred in males, whereas other etiologies, including cardiac surgery, CHD, myocarditis, and immune disease, most frequently occurred in females. Notably, AVB with unknown etiology accounted for more than one-third of the patients in both sex groups, although it was more common in females (46.1%) than males (34.2%) (**Supplementary Fig. 2**).

### 3.2 Clinical Profiles of Patients with Known and Unknown Etiologies

Clinical profiles of young AVB patients are presented in Table 1. In sum, patients with unknown etiologies had fewer symptoms of syncope, dizziness, and fatigue (all *p* < 0.01). Patients with undetermined etiologies exhibited fewer comorbidities than did those with determined etiologies, including coronary artery disease, valve disease, pulmonary artery hypertension, myocarditis history, and chronic kidney disease. However, the New York Heart Association (NYHA) functional class was more normal in those with undetermined etiologies than in those with determined etiologies. There is no statistical significance in the mild or advanced AVB proportion between the two groups (*p >* 0.5). Overall, patients with known etiologies exhibited a significantly lower heart rate than did those with unknown etiologies (Known: 62 [44–74] bpm *vs* Unknown: 56 [43–71] bpm; *p* = 0.042) (Table 1). Patients with known etiologies also had a higher proportion of right bundle branch block and non-sustained ventricular tachycardia, as well as a more prevalent change in cardiac structure and function than did their counterparts in the unknown group. Levels of NT-proBNP on admission were significantly higher in patients with known etiologies (Known: 495.0 [103–1702.4] pg/mL; Unknown: 70.2 [26–312.6] pg/mL; *p* < 0.001). However, no statistically significant differences were observed between the groups with regard to creatine kinase levels (Table 1).

**Fig. 3.**
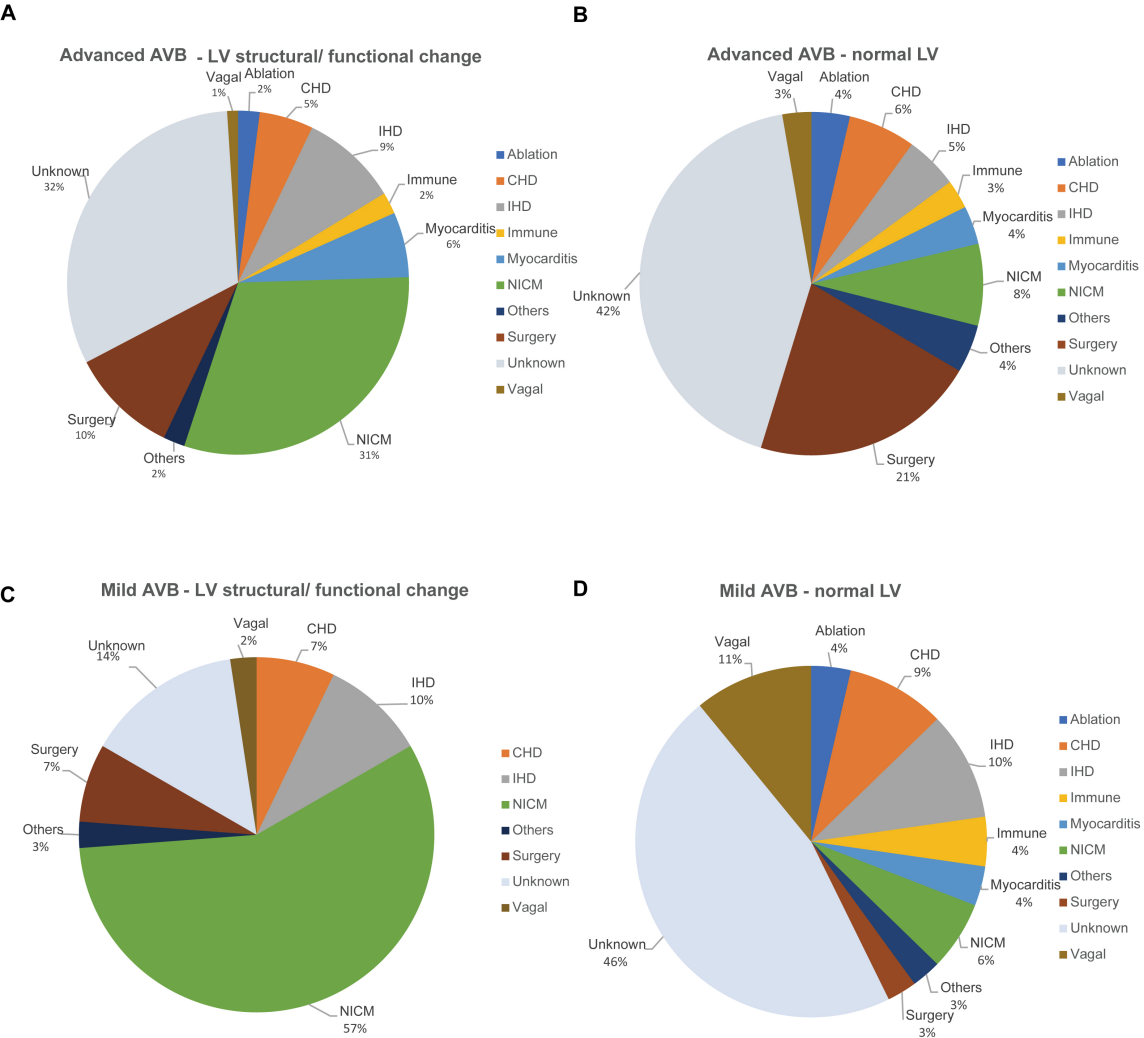
**AVB severity and left ventricle structural or functional change in relation to different etiologies**. (A) Etiology distribution in the young patients with advanced AVB and LV structural/functional change. (B) Etiology distribution in the young patients with advanced AVB but normal LV structure and function. (C) Etiology distribution in the young patients with mild AVB and LV structural/functional change. (D) Etiology distribution in the young patients with mild AVB and normal LV structure and function. Advanced AVB: Mobitz Type II AVB, high-degree AVB, or third-degree AVB. LV structural/functional change: abnormal left ventricular ejection fraction borderline/elevated left ventricular end diastolic diameter. AVB, atrioventricular block; LV, left ventricle; CHD, congenital heart disease; IHD, ischemic heart disease; NICM, non-ischemic cardiomyopathy; others comprise the etiologies with percentage lower than 1%, including AVB related to infiltrative cardiomyopathy, neuromuscular disease, long QT or Brugada syndrome, congenital AVB, endocrine or hereditary causes.

Previous studies have reported that Mobitz II, high-degree, and third-degree AVB routinely compromise the major pacing indication in AVB patients, and suggested that left-ventricle structure and function potentially affect a patient’s prognosis even after pacing therapy [1, 30, 31]. Therefore, we analyzed the etiologic distribution targeting AVB severity as well as left ventricle structural and functional change. Profiles of AVB severity and LV change across different etiologies are illustrated in Fig. 3. Although we found heterogeneity across groups, with regard to AVB severity and LV change, there were typical features of etiology distribution in different conditions. Specifically, NICM was the predominant etiology in those with LV structural or functional change with advanced AVB. On the other hand, vagal-mediated AVB accounted for the highest proportion of etiology in mild AVB with normal LV. Besides, AVB patients with unknown etiologies constitute a high proportion of those having advanced AVB with and without LV change (Fig. 3).

### 3.3 Clinical Phenotypes and Pacing Outcomes of Patients with Unknown Etiologies 

**Fig. 4.**
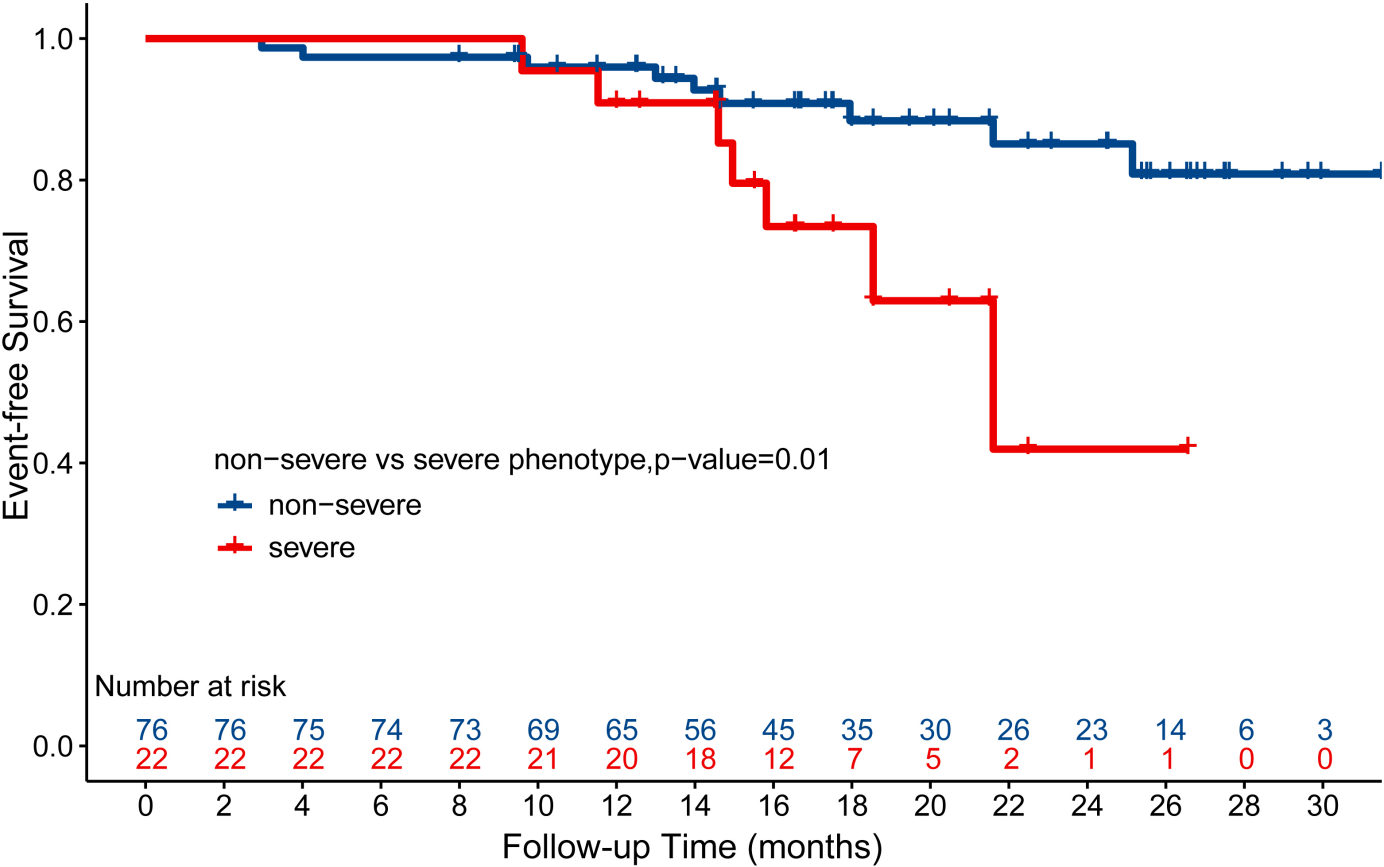
**Kaplan-Meier survival curves of event-free survival stratified by severe and non-severe clinical phenotypes among pacemaker recipients with unknown etiology**. Patients with complete follow-up information were included for analysis. Heart failure events include new onset of heart failure-related symptoms and signs and unplanned hospitalization due to the symptoms. Log-rank test was applied for survival rate comparison.

Considering the significant distribution of the unknown etiology group in the above conditions, we further explored the clinical characteristics of 182 AVB patients with unknown etiologies. We found significant differences in demographic features across the four groups; the structural change-dominated phenotype and mild phenotype were in younger and more likely male patients. There were no statistically significant differences in comorbidities, except for a high proportion of obstructive sleep apnea-hypopnea syndrome in the mild phenotype. Patients with severe phenotypes accounted for 17.03% of the undetermined etiology group, they tended to exhibit lower heart rates and a higher proportion of third-degree AVB than did those with other phenotypes (Table 2). A total of 110 (out of the 182) patients finally received pacing therapy. Complete follow-up was achieved in 98 of them, with 4 refusing to consent to follow-up and 8 losing follow-up. We found no statistically significant differences in baseline features between the total (n = 110) and just those with complete survival information (**Supplementary Table 2**). Analysis of the aforementioned four phenotypes revealed that 25 of the patients exhibited the severe phenotype, and 75 with the advanced AVB phenotype were pacemaker recipients. On the other hand, only 8 and 2, respectively, of the patients in the mild and LV change-dominated groups received pacing therapy (**Supplementary Table 2**). We recorded no deaths at a median follow-up of 17.5 months, but 16 patients reported (at least once) new-onset of HF symptoms. Among them, 2 were hospitalized because of the symptom. In 64 (13 with susptected HFEs, 51 without suspected HFEs) of the 98 (65.3%) patients whose follow-up echocardiographic results were also aviliable, we compared LVEDD and LVEF alteration in those with and without suspected HFEs. There is significant increase in LVEDD (*p* = 0.0249) and reduction in LVEF (*p* < 0.001) between baseline and follow-up in those with suspected HFEs (**Supplementary Fig. 3A,B**), while among those without suspected HFEs, the follow-up LVEDD and LVEF did not differ significantly between the baseline and follow-up echocardiography (**Supplementary Fig. 3C,D**), indicating that the symptoms/sign-based HFE event collected is in parallel with echocardiographic change. Kaplan-Meier survival curves showed that patients with severe phenotype had significantly higher suspected HFE rates than did their counterparts in the less severe phenotype group (log-rank *p* = 0.01) (Fig. 4). The Cox proportional hazard model yielded an unadjusted hazard ratio (HR) of 3.306, with minimally and fully adjusted HRs of 3.382 and 3.457, respectively. The logistic regression model also yielded similar results, as evidenced by an unadjusted odds ratio (OR) of 3.474, as well as minimally and fully adjusted ORs of 3.558 and 4.562, respectively (Table 3). Intriguingly, among the 22 patients with severe phenotype, we observed a trend of higher event rates in those who received traditional RV pacing (54.5%, n = 11) than among those under physiology pacing therapy (9.1%, n = 11) (log-rank *p* = 0.07) (**Supplementary Fig. 4**). Collectively, these results indicated that the severe phenotype is associated with a higher risk of HF symptoms after pacing therapy.

**Table 2.** **Clinical phenotypes of AVB with underdetermined etiology**.

Variables			Mild phenotype (n = 51)	AVB dominant (n = 94)	LV structural change dominant (n = 6)	Severe phenotype (n = 31)	*p*-value
Age, yrs			31.82 ± 11.88	36.76 ± 11.87	25.83 ± 14.23	36.90 ± 9.38	0.016
Male sex, n %			34 (66.67%)	44 (46.81%)	5 (83.33%)	16 (51.61%)	0.060
BMI, kg/$m^{2}$			24.14 ± 4.84	23.16 ± 4.01	22.60 ± 2.34	24.99 ± 4.28	0.161
Asymptomatic, n %			17 (33.33%)	8 (8.51%)	1 (16.67%)	3 (9.68%)	<0.001
Onset age, yrs			30.90 ± 11.92	32.54 ± 13.86	24.67 ± 14.90	32.68 ± 11.73	0.480
Family history, n %			4 (7.84%)	6 (6.38%)	0 (0.00%)	2 (6.45%)	0.905
Comorbidities							
	Coronary artery disease, n %		3 (5.88%)	7 (7.45%)	0 (0.00%)	0 (0.00%)	0.414
	Valve disease, n %		5 (9.80%)	5 (5.32%)	0 (0.00%)	3 (9.68%)	0.620
	Pulmonary artery hypertension, n %		3 (5.88%)	9 (9.57%)	2 (33.33%)	5 (16.13%)	0.130
	Stroke, n %		0 (0.00%)	2 (2.13%)	0 (0.00%)	2 (6.45%)	0.273
	OSAHS, n %		9 (17.65%)	2 (2.13%)	0 (0.00%)	0 (0.00%)	<0.001
	Diabetes, n %		4 (7.84%)	6 (6.38%)	0 (0.00%)	2 (6.45%)	0.905
	Myocarditis, n %		0 (0.00%)	1 (1.06%)	0 (0.00%)	1 (3.23%)	0.588
	Thyroid disease, n %		2 (3.92%)	5 (5.32%)	0 (0.00%)	0 (0.00%)	0.566
	Hypertension, n %		8 (15.69%)	13 (13.83%)	0 (0.00%)	8 (25.81%)	0.295
	Heart rate, bpm		70.00 (62.50–75.00)	51.00 (40.25–64.50)	62.50 (61.25–78.75)	43.00 (40.00–50.00)	<0.001
	Persistent AVB, n %		2 (3.92%)	36 (38.30%)	0 (0.00%)	16 (51.61%)	<0.001
Other ECG features							
	Atrial fibrillation/flutter, n %		5 (9.80%)	12 (12.77%)	1 (16.67%)	7 (22.58%)	0.419
	LBBB, n %		0 (0.00%)	9 (9.57%)	1 (16.67%)	2 (6.45%)	0.114
	RBBB, n %		3 (5.88%)	9 (9.57%)	0 (0.00%)	2 (6.45%)	0.736
	Non-sustained VT, n %		3 (5.88%)	5 (5.32%)	1 (16.67%)	3 (9.68%)	0.624
	NYHA class		1.1 ± 0.4	1.1 ± 0.4	1.5 ± 0.8	1.5 ± 0.7	<0.001
		I, n %	46 (90.2%)	87 (92.6%)	4 (66.7%)	21 (67.7%)	0.011
		II, n %	4 (7.8%)	5 (5.3%)	1 (16.7%)	6 (19.4%)	
		III, n %	1(2.0%)	2 (2.1%)	1 (16.7%)	4 (12.9%)	
		IV, n %	0 (0.0%)	0 (0.0%)	0 (0.0%)	0 (0.0%)	
	Left atrial diameter, mm		32.37 ± 5.09	34.19 ± 6.22	39.17 ± 10.17	39.90 ± 6.06	<0.001
	Left ventricular end diastolic diameter, mm		46.49 ± 4.57	47.04 ± 4.31	60.33 ± 14.84	57.52 ± 4.53	<0.001
	Left ventricular ejection fraction, %		64.80 ± 5.00	62.96 ± 4.66	54.67 ± 11.66	59.71 ± 8.17	<0.001
	Right ventricular diameter, mm		23.14 ± 5.65	22.93 ± 3.69	22.67 ± 2.42	24.61 ± 3.90	0.295
	Abnormal TAPSE, n %		2 (3.92%)	2 (2.13%)	0 (0.00%)	0 (0.00%)	0.673
	NT-proBNP, pg/mL		29.00 (14.05–82.05)	72.60 (33.12–429.52)	90.00 (34.25–244.75)	147.70 (85.45–471.50)	<0.001
	CK, IU/L		83.00 (56.00–110.50)	71.50 (56.50–98.75)	68.00 (60.50–80.75)	83.00 (61.50–113.00)	0.438

AVB, atrioventricular block; LV, left ventricle; BMI, body mass index; OSAHS, obstructive sleep apnea-hypopnea syndrome; ECG, electrocardiogram; LBBB, left bundle branch block; RBBB, right bundle branch block; VT, ventricular tachycardia; TAPSE, tricuspid annular plane systolic excursion; NYHA, the New York Heart Association; NT-proBNP, N-terminal B-type natriuretic peptide; CK, creatine kinase. The continuous parameters were displayed as mean ± standard deviation or median (25%, 75% interquartile).

**Table 3.** **Association of severe phenotype and composite outcomes among pacemaker recipients in young AVB patients with unknown reason**.

		Severe phenotype	Non-severe phenotype
Median follow-up duration, months		16.54	17.52
Number, n		22	76
Events, n		7	9
Cox regression			
	HR unadjusted	3.31	ref
	95% CI	1.20–9.12	
	*p*-value	0.021	
	HR minimally adjusted ^a^	3.38	ref
	95% CI	1.20–9.51	
	*p*-value	0.021	
	HR fully adjusted ^b^	3.46	ref
	95% CI	1.10–10.84	
	*p*-value	0.033	
Logistic regression			
	OR unadjusted	3.47	ref
	95% CI	1.12–10.81	
	*p*-value	0.032	
	OR minimally adjusted ^a^	3.56	ref
	95% CI	1.10–11.50	
	*p*-value	0.034	
	OR fully adjusted ^b^	4.56	ref
	95% CI	1.11–18.38	
	*p*-value	0.033	

a. Adjusted for age and sex. b. Adjusted for age, sex, NYHA classes II-III, CAD, PH, valve, log (NT-proBNP), pacing type and LBBB. AVB, atrioventricular block; HR, hazard ratio; OR, odds ratio; CI, confidence interval; ref, represent that non-severe phenotype was set as a reference in the regression model.

## 4. Discussion

In this study, we first comprehensively described the etiologic distribution and clinical features of AVB in a Chinese sample of patients under 50 years of age. Our results revealed various etiologies that cause AVB with distinct age- and sex-specific distributions, of which NICM and complications from cardiac surgery were the top known etiologies. Notably, the specific cause of AVB could not be determined in 38.6% of the patients, which indicates that there is still a large gap in etiologic diagnoses in this group. Next, we summarized distinct clinical features observed in AVB patients with unknown etiologies, targeting AVB severity and LV change. Results showed that patients with severe phenotype featuring advanced AVB and abnormal LV structure/function were associated with a significantly higher risk of HF symptoms, even after pacing therapy, than did their counterparts with less severe phenotypes.

The high AVB proportions in underdiagnosed patients observed in this study are consistent with findings from a previous larger registry from Denmark, which reported a higher proportion (50.3%) [9]. The discrepancy between studies might be due to differences in the study period and population. In the Denmark registry study, the authors analyzed etiology based on an earlier period (between 1996 and 2015), whereas our study was based on clinical data obtained between 2019 and 2022, a period when the guidelines about diagnostic tests of bradycardia, including imaging and laboratory tests, have markedly advanced. It should be noted that there is also difference in the study population. The Danish registry study mainly focused on etiologic distribution among pacemaker receivers, in which third-degree AVB dominate the study population. In comparision ,we analyzed the etiology in consecutive AVB patients both with and without pacemaker implantation, with advanced or third-degree AVB accounting for only 57.7% of the study population. Therefore, the seemingly low rate of syncope or amaurosis was observed in our study. But the analysis was restricted to the high-degree and third degree AVB subgroup (n = 272), these symptoms were still commonly seen in 30.5% patients as previously reported [9].

Analysis of AVB etiology revealed a specific difference between sexes. In sum, NICM, IHD, neuromuscular disease, and vagal-mediated AVB were the dominant AVB causes in men, whereas cardiac surgery, CHD, myocarditis, and immune disease were the main causes in women. A similar distribution pattern was also observed in sex distribution of the etiologies themselves, therefore, the patterns can provide clues for etiologic screening across different sex groups upon detection of AVB onset. Moreover, premature IHD should also be considered and checked at the time of AVB diagnosis in this group owing to the distinctly high proportion of IHD-mediated AVB in patients aged 36–50 years. Analysis of the relationship between etiology and AVB severity and LV structure/function revealed that AVB due to cardiac surgery accounts for a higher proportion in the advanced AVB without LV change phenotype. By contrast, as a leading cause of AVB in young patients, NICM is a progressive disease and a major cause of AVB, and tends to be accompanied by advanced AVB and LV change, which should raise concern. Previous studies have reported that NICM patients exhibit a high burden of various AVBs, which may also serve as the primary manifestation before onset of cardiac dysfunction [32, 33]. AVB patients with NICM exhibited significant changes in LV structure and function, with approximately 60% having advanced AVB. Importantly, the previously reported lower rate of NICM-related AVB in pacemaker receivers mainly included those with advanced AVB. Previous studies have also demonstrated that even first-degree AVB is not as benign as expected, and not only serves as an early marker but also as an indicator for poor prognosis [34]. Therefore, we recommend that NICM be considered as an etiology during diagnosis of AVB in young patients, and comprehensively evaluated using advanced laboratory or imaging tests.

AVB patients with unknown etiologies exhibited a low rate of abnormal LVEF, better NYHA class, and lower NT-proBNP levels than did their counterparts with known etiologies. A previous study reported that AVB patients younger than 50 with unknown etiologies also displayed a high risk of poor prognosis even under pacing therapy, indicating that AVB with unknown etiologies also might not be as benign as expected [10]. Results from the present study showed that although etiologies with pronounced phenotype and clear histories, such as surgery, CHD, IHD, or ablation-induced AVB, can be generally ruled out of the patients’ unknown etiologies, particular diseases such as hereditary AVB, infiltrative CM, NICM, and neuromuscular disease can be mixed in with undetermined etiologies. This can be restricted by either the preclinical stage of the disease or the lack of detailed testing approaches. After all, besides the ECG, echocardiography, and myocardial biomarker tests, the overall proportion of advanced special tests were low: molecular-genetic testing (1.6%), myocardial biopsy (0.5%), cardiovascular magnetic resonance (13.2%), positron emission/ single-photon emission computed tomography imaging (3.3%), or antibody test (11%). We also speculate that a lack of these particular tests might be responsible for misidentification of the proportion of corresponding etiologies, whereas the actual proportion of those systematic, progressive, rare but latent etiologies, might be higher than presented herein.

Even in the absence of clear etiologies in primary care, clinicians should also consider identifying the potential malignant phenotype that might affect patient prognosis. Based on the previously mentioned AVB severity and LV change, AVB patients with unknown etiologies were divided into four types with distinctive characteristics, of which 17% showed risk features such as advanced AVB, increased LVEDD, and high NT-proBNP levels [35, 36] and were classified as the severe phenotype. Among AVB patients with undetermined etiologies who received pacemakers, those who presented with severe phenotype were associated with a triple risk of developing HF symptoms and signs. In the same subgroup, those who received traditional RV pacing (n = 11) exhibited a higher rate of suspected HFEs than did those in the physiological pacing (1 CRT and 10 left bundle branch area pacing) group (54.5% *vs* 9.1%). Although this clinical phenotype cannot substitute for a definitive etiologic diagnosis for AVB, these observations are of clinical importance as they will guide future characterization of the patients’ risk even if the cause is obscure and the overall LVEF is normal (59.7 ± 8.2%). In fact, the severe type mainly displayed pronounced borderline or increased LVEDD, which was previously described as a sensitive predictor of adverse cardiac events in patients with LVEF above 50% and an indicator of future LVEF change [36]. Therefore, such a structural manifestation might represent early impairment of the heart. However, the optimal therapy, if any, for these individuals remains uncertain. We observed a beneficial trend in patients who received a physiological pacing strategy compared to those in the traditional RV pacing group, although the result was not fully adjusted due to the limited sample size. We suspect that progression of the undetermined disease, coupled with the desynchrony induced by RV pacing might contribute to the higher rate of suspected HFEs observed in the RV pacing group, although biventricular pacing or conduction system pacing, which provide better electrical and mechanical cardiac synchrony, might be unlikely to cause deterioration of the condition.

## 5. Limitations

Considering that this was a retrospective study, although we predetermined the diagnostic work-up and data collection spectrum, and included those with complete information, the same well-defined testing series cannot be guaranteed during the real diagnosis phase. Diagnosis of some etiologies may also be restricted by the lack of a key diagnostic test, thereby resulting in a lower proportion of some rare diseases. Another limitation was the exclusion of 7% of patients due to a lack of medical records. However, we found no statistically significant differences in demographic features and comorbidities between enrolled and excluded cases. In addition, the suspected HFE was mainly based on signs and symptoms rather than on objective measurements during follow-up of pacemaker recipients with unknown AVB reasons due to the high proportion of missing echocardiographic results during follow-up. But in the 65.3% patients with both symptom/signs evaluation and echocardiographic results, the suspected HFEs were in parallel with LVEDD and LVEF alteration. And previous studies have shown agreement between the self-reported HF symptoms and objective medical evaluation [37]. Finally, the small number of events observed limited multivariate analysis, and there might be the possibility of overfitting after adjusting for multiple confounders. Considering these limitations, there should be caution when interpreting these findings: this study was for description and hypothesis generation rather than seen as definitive claims. Further long-term follow-up for hard clinical endpoints and results from prospective studies are required.

## 6. Conclusions

The etiologies of AVB in young inpatients are diverse but still underdiagnosed. The etiology distribution has age- and gender-specific patterns. As a progressive condition, NICM is a leading cause of AVB characterized by predominate LV structural or functional change, which warrants attention upon clinical diagnosis of AVB. And among patients with unknown AVB etiologies, those with severe phenotype with a change of LV structure and advanced AVB were at a relatively higher risk of suspected HF symptom onset despite pacing therapy, and deserved further investigation. These findings provide new insights into the clinical characteristics and complexity of AVB in young AVB patients and emphasize the need for etiology diagnosis. Future studies are needed to raise the etiologic diagnosis rate, investigate the contribution of etiology to prognosis, and explore the appropriate pacing strategies for young AVB patients.

## Data Availability

The datasets supporting this work are available from the corresponding author on reasonable request.

## Abbreviations

AVB, atrioventricular block; BMI, body mass index; OSAHS, obstructive sleep apnea-hypopnea syndrome; LVEF, left ventricular ejection fraction; LVEDD, left ventricle diastolic diameter; NICM, non-ischemic cardiomyopathy; HF, heart failure; CIED, cardiac implantable electronic device.

## Supplementary Material

Supplementary material associated with this article can be found, in the online version, at https://doi.org/10.31083/j.rcm2409250.
