# Supplementary material for: Etiology Distribution, Clinical Characteristics, and Suboptimal Pacing Outcome of Atrioventricular Block in Young Patients
Source: Rev Cardiovasc Med. 2023 Sep 6;24(9):250. doi: 10.31083/j.rcm2409250 (PMC11270075; doi:10.31083/j.rcm2409250)
Supplement: Supplementary file 1 [file 2153-8174-24-9-250-s1.zip › 2153-8174-24-9-250-s1.docx]

**
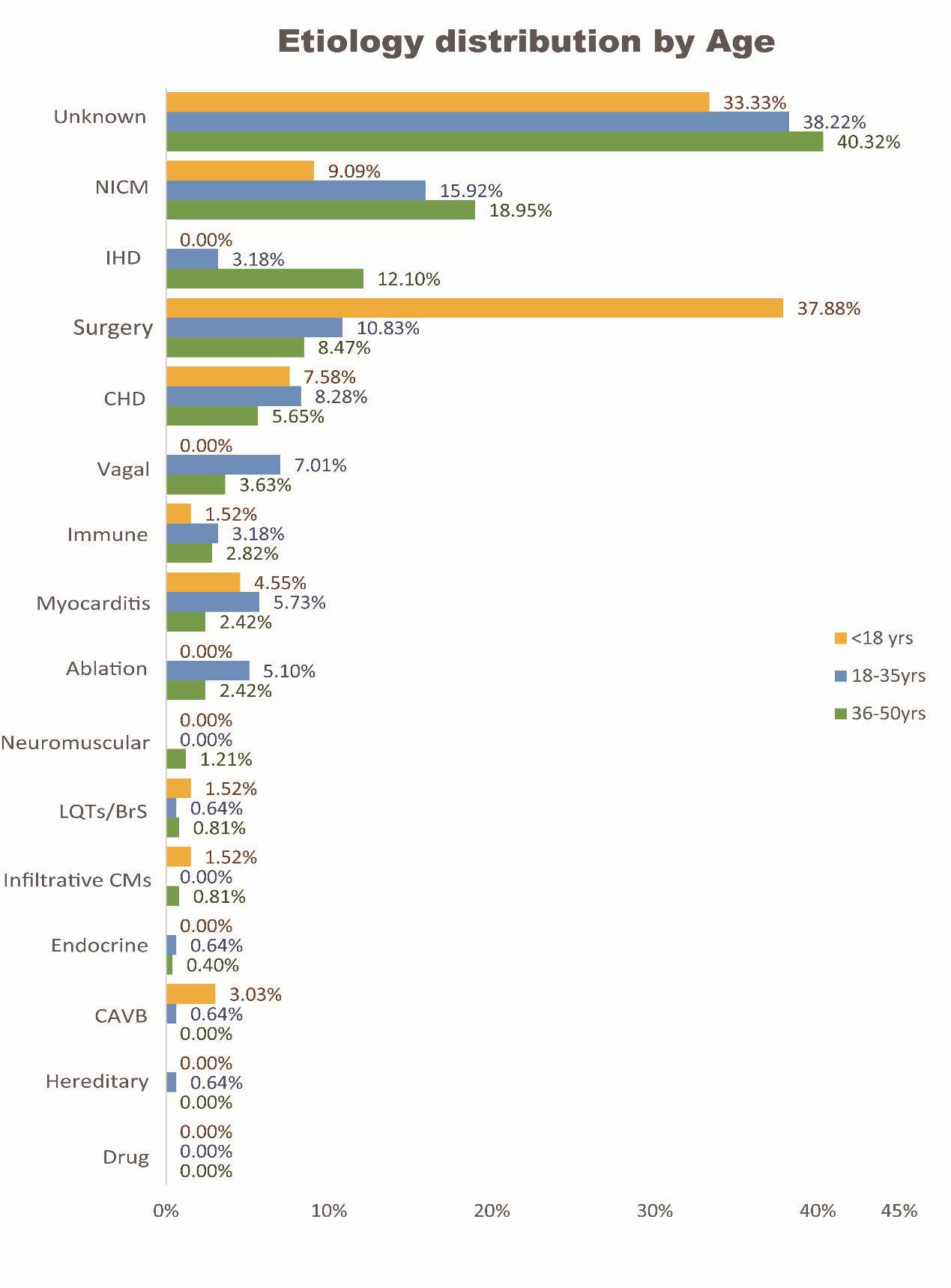
**

Supplementary Fig.1. Age-specific etiological distributions of AVB in patients ≤50 years.

CAVB, congenital atrioventricular block; CHD, Congenital heart disease; NICM, non-ischemic cardiomyopathy; LQTs, long QT syndrome; BrS, brugada syndrome; IHD, ischemic heart disease. Distribution of etiology between the three age groups, p<0.001.

**
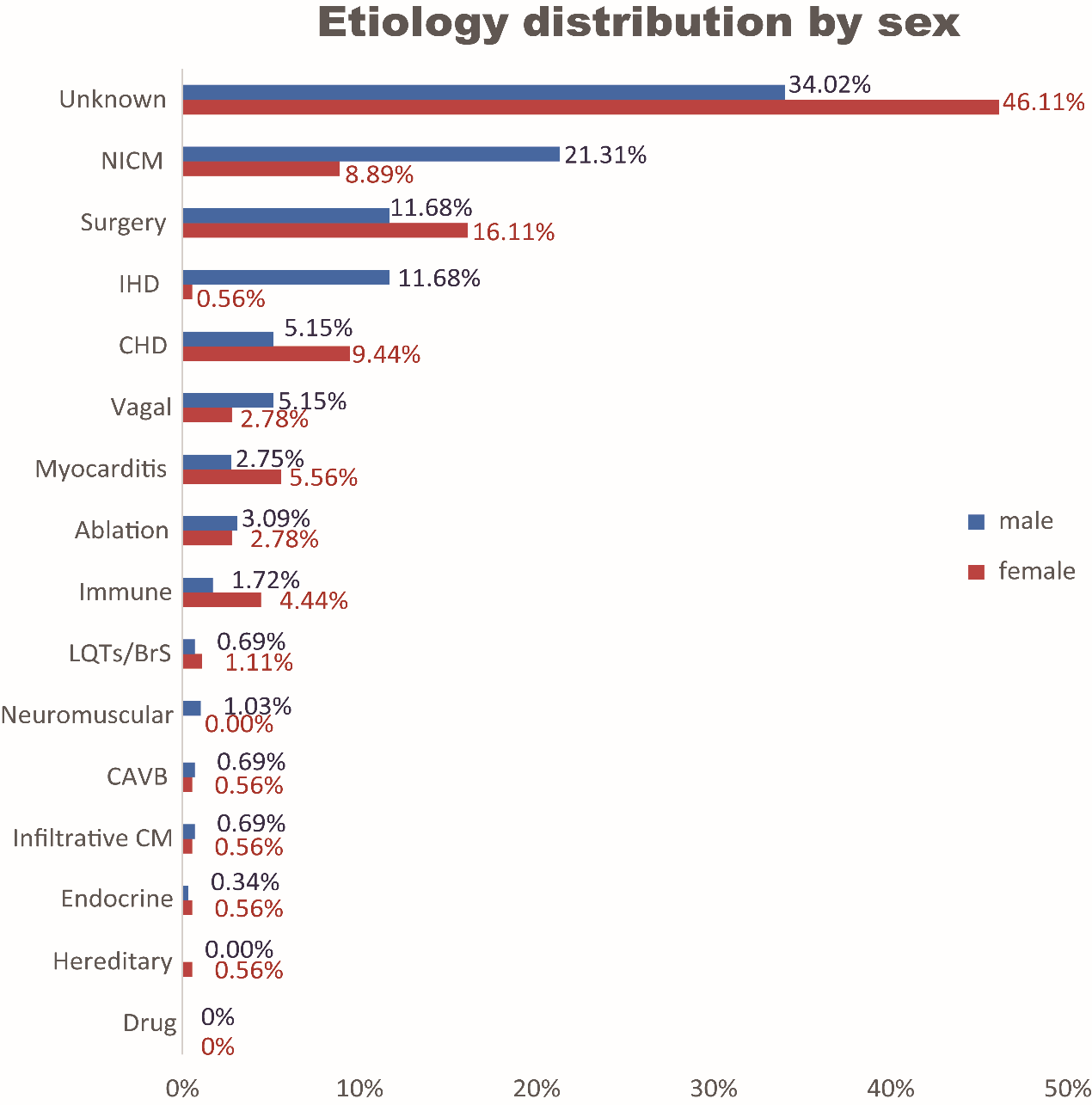
**

Supplementary Fig. 2. Sex-specific etiological distributions of AVB in patients ≤50 years.

CAVB, congenital atrioventricular block; CHD, congenital heart disease; NICM, non-ischemic cardiomyopathy; LQTs, long QT syndrome; BrS, Brugada syndrome; IHD, ischemic heart disease. Distribution of etiology between male and female groups, p<0.001.

**
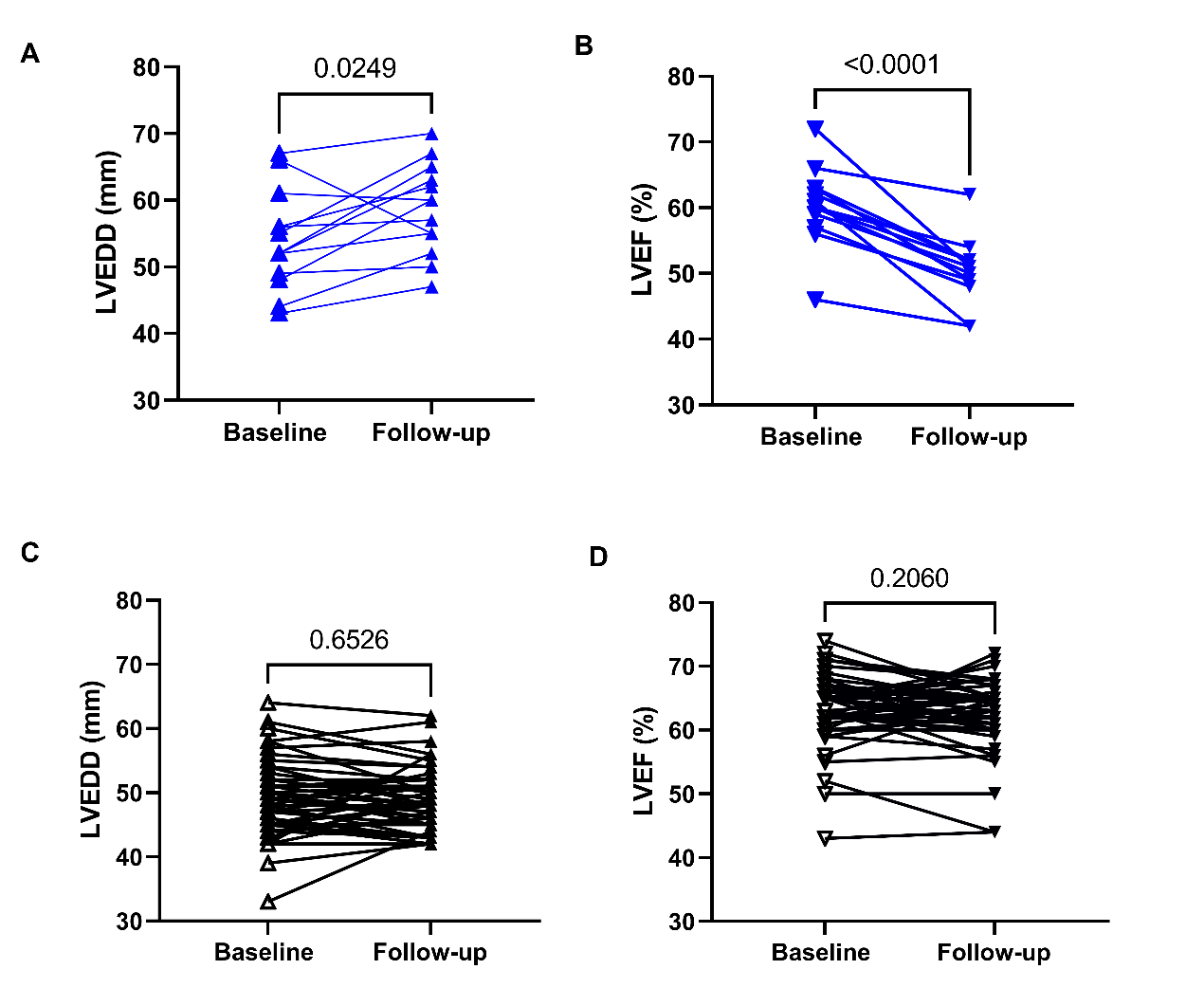
**

Supplementary Fig. 3. Change of LVEF and LVEDD in patients with both follow-up of both HF symptoms/signs and echocardiography evaluation.

A-B: Baseline and follow-up LVEF and LVEDD in patients with suspected HFEs.

C-D: Baseline and follow-up LVEF and LVEDD in patients without suspected HFEs.

Suspected heart failure events include new onset of heart failure-related symptoms and signs and unplanned hospitalization due to the symptoms.

**
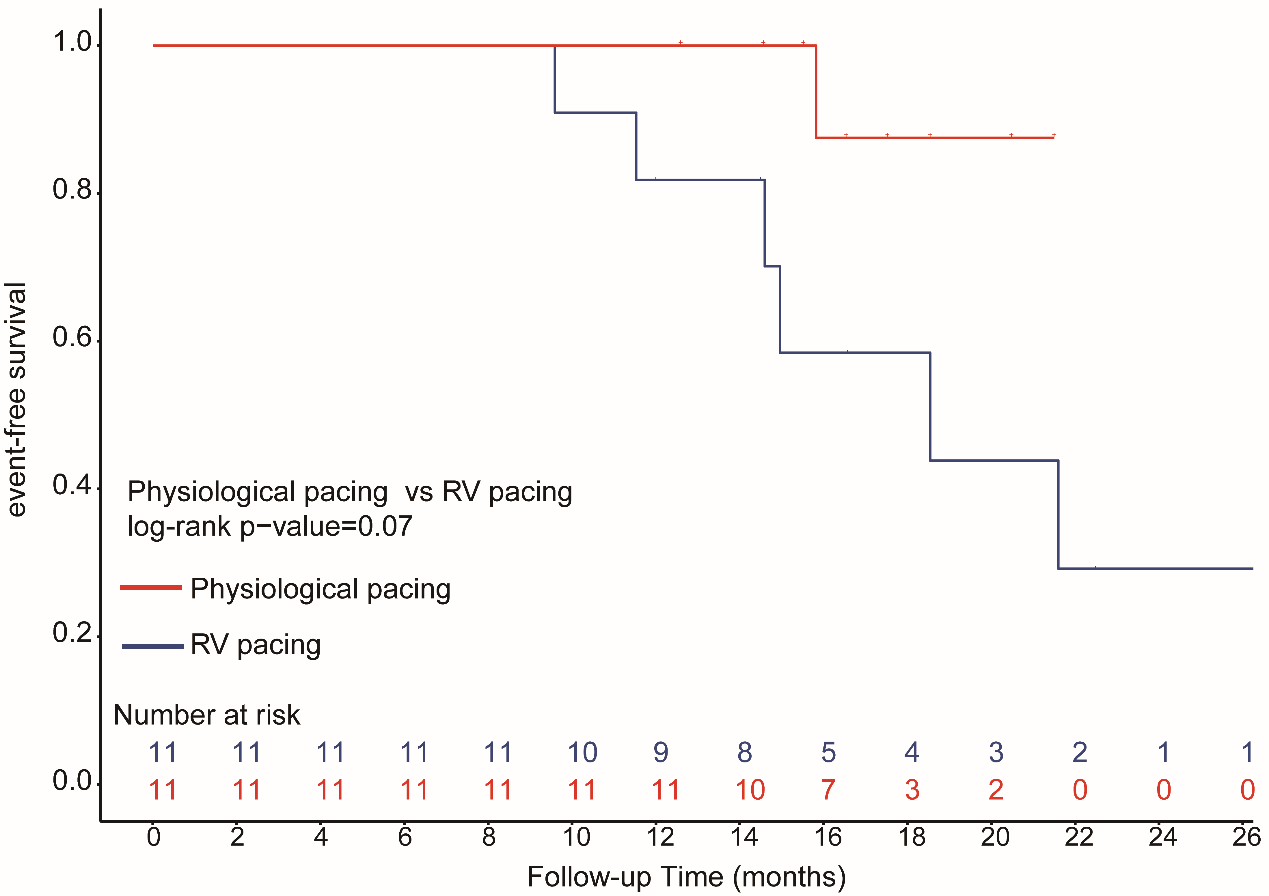
**

Supplementary Fig. 4. Kaplan-Meier survival curves of event-free survival stratified by right ventricular pacing and physiological pacing among the severe phenotype subgroup in pacemaker recipients with unknown etiology.

Suspected heart failure events include new onset of heart failure-related symptoms and signs and unplanned hospitalization due to the symptoms. Log-rank test was applied for survival rate comparison.

**Supplementary Table 1. Baseline characteristic and etiology of AVB in pediatric patients.**

| **Parameters** | **Pediatric patients (n=66)** |
| --- | --- |
| **Age, years** | 10.1±6.0 |
| **Age categories according**  **to NICHD Pediatric Terminology** | |
| Infancy (< 1 year), n % | 5 (7.6%) |
| Toddlerhood (1-2 years), n % | 8 (12.1%) |
| Early childhood (3-5 years), n % | 8 (12.1%) |
| Middle childhood (6-11 years), n % | 8 (12.1%) |
| Early adolescence (12-17 years), n % | 37 (56.1%) |
| **Sex** |  |
| Female, n % | 30 (45.5%) |
| Male, n % | 36 (54.5%) |
| **Etiology of AVB** |  |
| Congenital AVB, n % | 2 (3.0%) |
| Congenital heart disease, n % | 5 (7.6%) |
| NICM, n % | 6 (9.1%) |
| Immune disease, n % | 1 (1.5%) |
| Myocarditis, n % | 3 (4.5%) |
| Infiltrative cardiomyopathy, n % | 1 (1.5%) |
| Brugada syndrome, n % | 1 (1.5%) |
| Complication of cardiac surgery, n % | 25 (37.9%) |
| Underdiagnosed etiology, n % | 22 (33.3%) |
| **AVB type** |  |
| Mild AVB, n % | 20(30.3%) |
| Advanced AVB, n % | 46(69.7%) |
| Mobitz type II, | 7 (10.6%) |
| High degree AVB | 35 (53.0%) |
| Third degree AVB | 4 (6.1%) |
| **CIED implantation，**n % | 39 (59.1%) |
| Pacemaker，n % | 38 (57.6%) |
| Physiological pacing, n % | 5 (7.6%) |
| Traditional RV pacing, n % | 33 (50.0%) |
| ICD, n % | 1 (1.5%) |

AVB, atrioventricular block; NICHD, Eunice Kennedy Shriver National Institute of Child Health and Human Development; NICM, non-ischemic cardiomyopathy; CIED, cardiac implantable electronic device; RV pacing, right ventricular pacing; ICD, implantable cardiac defibrillator.

**Supplementary Table 2. Comparison of Baseline characteristics of young patients with unknown AVB reason who received pacing therapy and who have complete follow-up data**

| **Variables** | Pacemaker recipients  n=110 | Pacemaker recipients with Complete follow-up  n=98 | *P-*value |
| --- | --- | --- | --- |
| Age, yrs | 36.4 ± 11.3 | 35.6 ± 11.5 | 0.632 |
| Male | 50 (45.5%) | 42 (42.9%) | 0.707 |
| BMI, kg/m2 | 23.5 ± 4.2 | 23.3 ± 4.1 | 0.783 |
| NT-proBNP, pg/mL | 97.7 (45.3-416.5) | 87.4 (42.7-370.0) | 0.662 |
| Heart rate, bpm | 48.0 (40.0-56.8) | 49.5 (40.2-59.0) | 0.800 |
| Left atrial diameter, mm | 35.3 ± 6.3 | 34.7 ± 5.9 | 0.475 |
| Left ventricular end diastolic diameter, mm | 49.2 ± 6.0 | 49.0 ± 6.2 | 0.753 |
| LVEF, % | 62.4 ± 5.8 | 62.8 ± 5.4 | 0.568 |
| Right ventricular diameter, mm | 23.1 ± 3.5 | 22.9 ± 3.5 | 0.811 |
| Abnormal TAPSE, n % | 1 (0.9%) | 1 (1.0%) | 1.000 |
| NYHA | 1.1 ± 0.4 | 1.1 ± 0.4 | 0.742 |
| I | 100 (90.9%) | 91 (92.9%) |  |
| II | 6 (5.5%) | 3 (3.1%) |  |
| III | 4 (3.6%) | 4 (4.1%) |  |
| Coronary artery disease, n % | 7 (6.4%) | 6 (6.1%) | 0.943 |
| Pulmonary artery hypertension, n % | 6 (5.5%) | 4 (4.1%) | 0.644 |
| Valve disease, n % | 9 (8.2%) | 8 (8.2%) | 0.996 |
| Stroke, n % | 2 (1.8%) | 2 (2.0%) | 1.000 |
| Cancer, n % | 2 (1.8%) | 2 (2.0%) | 1.000 |
| OSAHS, n % | 2 (1.8%) | 1 (1.0%) | 1.000 |
| Diabetes, n % | 6 (5.5%) | 6 (6.1%) | 0.837 |
| Hyperlipidemia, n % | 23 (20.9%) | 21 (21.4%) | 0.927 |
| Myocarditis, n % | 2 (1.8%) | 2 (2.0%) | 1.000 |
| Thyroid disease, n % | 4 (3.6%) | 1 (1.0%) | 0.373 |
| Hypertension | 16 (14.5%) | 13 (13.3%) | 0.790 |
| Atrial fibrillation | 15 (13.6%) | 11 (11.2%) | 0.600 |
| LBBB | 11 (10.0%) | 11 (11.2%) | 0.774 |
| RBBB | 10 (9.1%) | 8 (8.2%) | 0.812 |
| NS-VT | 5 (4.5%) | 3 (3.1%) | 0.725 |
| **Phenotype** |  |  | 0.999 |
| Mild | 8 (7.3%) | 7 (7.1%) |  |
| AVB dominant | 75 (68.2%) | 67 (68.4%) |  |
| LV structural change dominant | 2 (1.8%) | 2 (2.0%) |  |
| Severe | 25 (22.7%) | 22 (22.4%) |  |
| **Pacing type** |  |  | 1.000 |
| Physiological pacing | 65 (59.1%) | 58 (59.2%) |  |
| Traditional RV pacing | 45(40.9%) | 40 (40.8%) |  |

BMI, Body mass index; NYHA, New York Heart Association; NT-proBNP, N-terminal B-type natriuretic peptide; OSAHS, obstructive sleep apnea-hypopnea syndrome; LBBB, Left bundle branch block; RBBB, Right bundle branch block; NS-VT, non-sustained ventricular tachycardia; TAPSE, Tricuspid annular plane systolic excursion; LVEF Left ventricular ejection fraction; AVB, atrioventricular block; LV, left ventricle; RV, right ventricle Physiological pacing includes biventricular pacing and conduction system pacing. The continuous parameters were displayed as mean ± standard deviation or median (25%, 75% interquartile).
